# Sea whip coral *Leptogorgia virgulata* in the Mid-Atlantic Bight: Colony complexity, age, and growth

**DOI:** 10.7717/peerj.8372

**Published:** 2020-02-04

**Authors:** Rebecca P. Wenker, Bradley G. Stevens

**Affiliations:** Department of Natural Sciences, University of Maryland Eastern Shore, Princess Anne, MD, United States of America

**Keywords:** Sea whips, Coral growth, Mid-atlantic bight, Annuli, Von bertalanffy, Coral complexity, Coral ageing

## Abstract

Sea whip coral *Leptogorgia virgulata* are a common structural component of both natural and artificial hard-bottom reef habitats in the mid-Atlantic region and may serve as essential habitat for commercially valuable species. However, they are slow-growing, easily damaged, and especially vulnerable to damage by passive fishing gear such as pots and traps. Despite their potential importance, until recently, sea whips have been generally understudied in this region. We examined the colony complexity, length, age, and growth of sea whips from four artificial reef sites in the mid-Atlantic region to gain a better understanding of their biology in the area. There were no significant differences in the bifurcation (R_b_) and tributary to source (T/S) ratios between sites, with the R_b_ ≈3 for all sites, indicating similar complexity between sites. The total length distribution was 8.3 cm to 85.3 cm, and 50% of corals in the range of 34.2–56.4 cm. Age, estimated from annual growth ring counts, ranged from 2 to 15 y, with 50% of corals in the range of 6 to 8 y. The large proportion of middle-sized and middle-aged corals suggests episodic recruitment. Age-length keys showed the trend of age increasing with total coral length, and a von Bertalanffy growth model demonstrated size-dependent growth following the equation: E[L—t] (cm) = 86.1(1−e^−0.14(t−1.44)^). This is the first study providing such data for sea whips in the coastal mid-Atlantic region, and the baseline created will be a useful reference to study changes over time.

## Introduction

Cold-water corals are an important contributor to benthic habitat complexity on continental shelves and slopes, canyons, seamounts, oceanic banks, and ocean ridges (Freiwald et al., 2004). They have also been observed to colonize man-made structures, such as artificial reefs or shipwrecks (Steimle & Zetlin, 2000; Freiwald et al., 2004). These coral habitats often serve as biodiversity hotspots and are used by other species for numerous purposes, including nurseries, feeding and spawning grounds, and refuge sites (Freiwald et al., 2004; Foley et al., 2010; Watling et al., 2011; Baillon et al., 2012; Capezutto et al., 2018). However, these communities have been negatively impacted by fishing activities such as bottom trawling, bottom-set gillnets and longlines, pots, and traps (Van Dolah, Wendt & Nicholson, 1987; Freiwald et al., 2004; Watling et al., 2011; Schweitzer, Lipcius & Stevens, 2018), which can inflict structural damage to the coral or completely remove them from the seafloor.

In the Mid-Atlantic Bight (MAB), ranging from Massachusetts to North Carolina on the U.S. east coast, benthic habitats are primarily flat and homogenous topography composed of sand and mud bedforms. Within this region, hard-bottom reef habitats are scarce, patchy, and widely scattered (Steimle & Zetlin, 2000). Reef habitats vary in composition and include both natural rocky bottom and mud outcrops as well as anthropogenic structures such as shipwrecks, pipes, lost cargos, and cable cars that form artificial reefs (Steimle & Zetlin, 2000). Due to the relative infrequency of natural hard-bottom substrate, introduced or artificial reef habitat is most likely a significant source of habitat complexity. Both natural and artificial reef structures provide multi-dimensionality and can support biological communities that the surrounding soft-bottom habitat cannot, including mussels, crabs, lobsters, corals, sponges, and numerous fish species (Sedberry & Van Dolah, 1984; Steimle & Figley, 1996; Steimle & Zetlin, 2000; Fabrizio, Pessutti & Manderson, 2013; Ross et al., 2016; Diaz, Cutter & Able, 2003; Scharf, Manderson & Fabrizio, 2006). Due to the high utilization of natural and artificial reef habitat by fish species, mid-Atlantic reefs are often well-known and targeted by recreational and commercial fisheries (Steimle & Zetlin, 2000).

In the Delaware, Maryland, and Virginia (Delmarva) region of the MAB the sea whip *Leptogorgia virgulata* is a common component of natural and artificial hard-bottom reefs (Steimle & Zetlin, 2000; Cullen & Stevens, 2017; Schweitzer, Lipcius & Stevens, 2018; Schweitzer & Stevens, 2019). These corals can be found along the North American Atlantic coast, and have been observed at depths of 2–59 m (Bayer, 1961; Gotelli, 1988; Devictor & Morton, 2010; Packer et al., 2017). Sea whips are non-reef building corals, but have a stiffened axial skeleton and a 3-dimensional structure with branches arranged around a central axis, adding additional height to reef substrate (Bayer, 1961; Mitchell, Dardeau & Schroeder, 1993; Devictor & Morton, 2010). Growth rings are deposited into this axial rod and assumed to be annual, enabling age estimation (Grigg, 1974; Mitchell, Dardeau & Schroeder, 1993). Notably, the structural complexity provided by sea whips and the biotic community associated with them may make these octocorals a significant habitat for many commercially and recreationally valuable species (Van Dolah, Wendt & Nicholson, 1987; Ruppert & Fox, 1988; Steimle & Figley, 1996; Able & Fahay, 1998; Wicksten & Cox, 2011; Cullen & Stevens, 2017). These include species like black sea bass *Centropristis striata*, tautog *Tautoga onitis*, and lobster *Homarus americanus* in the Delmarva region, and snapper *Lutjanus* spp*.*, grouper *Epinephelus* spp*.*, and porgy *Calamus* spp*.* in the South Atlantic Bight (Van Dolah, Wendt & Nicholson, 1987; Steimle & Figley, 1996; Able & Fahay, 1998; Cullen & Stevens, 2017). Additionally, Schweitzer & Stevens (2019) found fish abundance to positively correlate with sea whip coverage on artificial Delmarva reef habitats, and that sea whips were the only biogenic structure in the study significantly related to fish abundance.

Several studies have also indicated that healthy sea whips produce a strong chemical defense system, preventing the attachment, settlement, and fouling by epibionts (Targett et al., 1983; Standing, Hooper & Costlow, 1984; Gerhart, Rittschof & Mayo, 1988; Clare et al., 1999). Sea whips in the Delmarva region show evidence of damage and overgrowth by fouling organisms (R Wenker and B Stevens, pers. obs., 2017 and 2018; Schweitzer, Lipcius & Stevens, 2018; Schweitzer & Stevens, 2019). This includes overgrowth by organisms such as mussels, bryozoans, ascidians, and sponges, as well as damaged and stripped tissue. The presence of overgrowth and fouling could suggest that the underlying coral tissue has been damaged or killed either by natural causes or impacts with fishing gear, and the coral’s chemical defense system impaired.

Despite their potential importance to commercially valuable fish and shellfish species, sea whips are generally understudied in the western Atlantic, and little is known about the local natural and artificial reefs nor the sea whip colonies that occupy them. No standard or baseline information exists for comparison in case of major changes to this habitat, whether caused by human or natural disturbance. For example, there is currently no information regarding growth rates, effects of damage or fouling on growth and mortality, or rates of recovery from damage.

In response to this lack of information, this study was undertaken to provide new insights into the biology of sea whip corals. The goals of this project were to determine colony complexity, age, and growth rates of sea whips from four artificial reef sites in the mid-Atlantic region, in order to gain a better understanding of reef ecology in this understudied region.

## Materials & Methods

### Study sites

**Figure 1:**
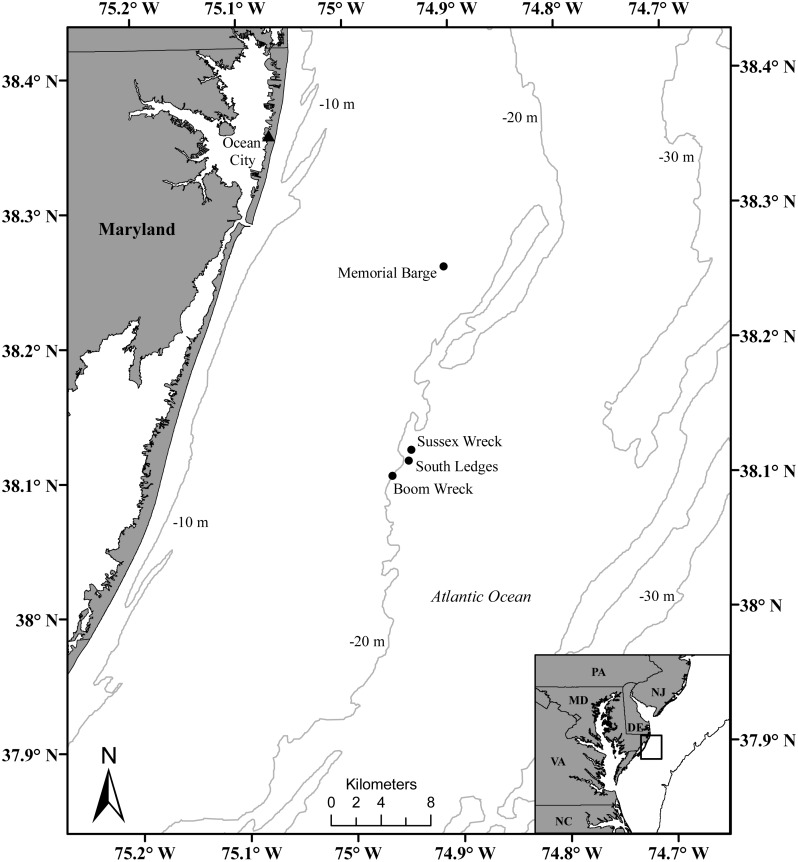
Map of the four artificial reef study sites offshore of Ocean City, MD, where corals were collected. Includes: Memorial Barge, South Ledges, Sussex Wreck, and Boom Wreck.

Sea whips were collected from four artificial hard-bottom reef sites located approximately 16 km offshore of Ocean City, MD (Table 1; Fig. 1). Depth differed between sites, with Memorial Barge being 17–20 m, South Ledges 17–21 m, Sussex Wreck 24–26 m, and Boom Wreck 21–24 m (Table 1). Memorial Barge was placed on the sea floor in 1993, South Ledges in 2000, Sussex Wreck in 1995, and Boom Wreck in an unknown year. Samples were taken from the Memorial Barge on October 3, 2016 and August 7, 2017, South Ledges on August 11, 2017, Sussex Wreck on August 10, 2018, and Boom Wreck on October 1, 2018.

### Sample measurement and collection

All sample collections were made via regular scuba diving. Though sea whips are not a managed species, approval for their collection was obtained from the National Oceanic and Atmospheric Administration. A total of 102 sea whips were collected; 24 from Memorial Barge, 26 from South Ledges, 29 from Sussex Wreck, and 23 from Boom Wreck (Table 1). At each site, two dives were conducted per each sampling day. On the first dive, scuba divers measured size frequency of the corals present using a systematic random sampling approach. This was done by stretching out a 50 meter tape measure from a random starting point within 1 m from the edge of the habitat along the longest dimension of the habitat, and selecting the specimen nearest to every 0.5 m mark along the tape. Coral colonies were stretched out, and their height (total length, TL) was marked with a pencil on a section of $ \frac{1}{2} $ inch diameter PVC pipe marked at one cm intervals.. A total of 119 corals were measured in-situ; 29 from Memorial Barge, 28 from South Ledges, 31 from Sussex Wreck, and 31 from Boom Wreck (Table 1). The length of the transect differed per site due to the varying size of each reef structure and abundance of sea whips, though we tried to stretch the tape across areas with higher densities of sea whips in order to achieve an acceptable sample size.

**Table 1:** Descriptive information of the four artificial reef study sites in the Mid-Atlantic Bight. Includes study site name, location, depth (m), date visited, number of corals measured in-situ (N_IS_) and number of corals collected (N_C_).

Study site	Latitude (decimal degrees, N)	Longitude (decimal degrees, W)	Depth (m)	Date	*N*_*IS*_	*N*_C_
Memorial barge	38.290983	−74.910817	17–20	10∕03∕2016	0	11
				08∕07∕2017	29	13
South ledges	38.151100	−74.946600	17–21	08∕11∕2017	28	26
Sussex wreck	38.159000	−74.944050	24–26	08∕10∕2018	31	29
Boom wreck	38.140550	−74.961767	21–24	10∕01∕2018	31	23

On the second dive, colonies were selected for estimation of age and colony complexity in a stratified manner. We attempted to sample a similar number of small, medium, and large colonies, based on the initial size frequency analysis. Specimens were collected by either removing the entire colony with its holdfast intact if possible, or by cutting the basal stalk at the point closest to the holdfast with bone cutters. Sampled colonies were placed in a large mesh bag for transport to the surface.

### Colony complexity

The total length of all collected coral specimens was measured to the nearest mm, and all branches were counted and labeled for branching analysis. Two measures of colony complexity were obtained for each collected colony: a bifurcation ratio (R_b_), and a tributary to source ratio (T/S).

**Figure 2:**
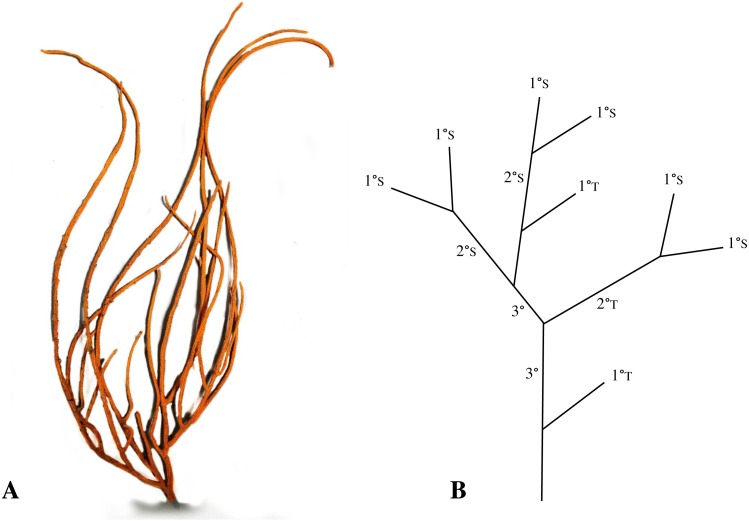
Photo of *Leptogorgia virgulata* (A), and an example of a simplified branching pattern (B). The branching pattern exhibits first (1°), second (2°), and third (3°) order branches, as well as their tributary (T) or source (S) status. Adapted from Brazeau & Lasker (1988).

The R_b_ is the ratio of the number of branches of a given order to the number of branches of the next higher order, a technique originally used to describe stream networks (Strahler, 1952; Brazeau & Lasker, 1988). Different branch levels were assigned, with the most distal branches being primary, the union of two primary branches forming a secondary, two secondary branches connecting to form a tertiary, etc (Fig. 2). The R_b_ is then obtained by regressing the log of number of branches versus branch order, and calculating the antilog of the slope of the regression line. Branching networks that display perfectly dichotomous branching have a R_b_ of 2, and this value increases as branches that do not increase the order of the system are added. A benefit to this ordering technique is that branches with similar functions are grouped in the same order (Mitchell, Dardeau & Schroeder, 1993; Brazeau & Lasker, 1988). For example, primary branches tend to have younger, non-reproductive polyps, whereas older, reproductive polyps are more frequently found on secondary and tertiary branches (Brazeau & Lasker, 1988). We calculated the R_b_ for each colony, and an average for each site.

While the R_b_ focuses primarily on overall colony complexity, the T/S ratio is more sensitive to differences at each level of branching. The T/S ratio distinguishes between branches which do or do not increase the order of the system (Mock, 1971). A branch that joins another branch of equal order is called a “source” branch, while branches that join a branch of higher order are “tributary” branches (Fig. 2) (Mock, 1971; Brazeau & Lasker, 1988). Following the methods of Brazeau & Lasker (1988) and Mitchell, Dardeau & Schroeder (1993), we calculated the T/S ratios of primary and secondary branches in each colony by dividing the number of tributary branches by the number of source branches. We then calculated an average primary and secondary T/S ratio per site.

The mean length of in-situ and collected corals were each compared between sites with a One-way ANOVA to test the null hypothesis of equality between sites. The R_b_ and T/S ratios of collected corals were each compared between sites with a One-way ANOVA (R function aov). Total length distributions of in-situ corals and collected corals were each compared between sites with two-sample Kolmogorov–Smirnov (KS) tests (R Core Team, 2017). Only two sites can be compared simultaneously with a KS test, so 6 tests were conducted to compare all the sites for both in-situ and lab measured corals. We adjusted the critical *P*-value (*α*) accordingly using the Bonferroni correction method, dividing 0.05 by 6 to get a new critical *α* of 0.008.

### Age analysis

**Figure 3:**
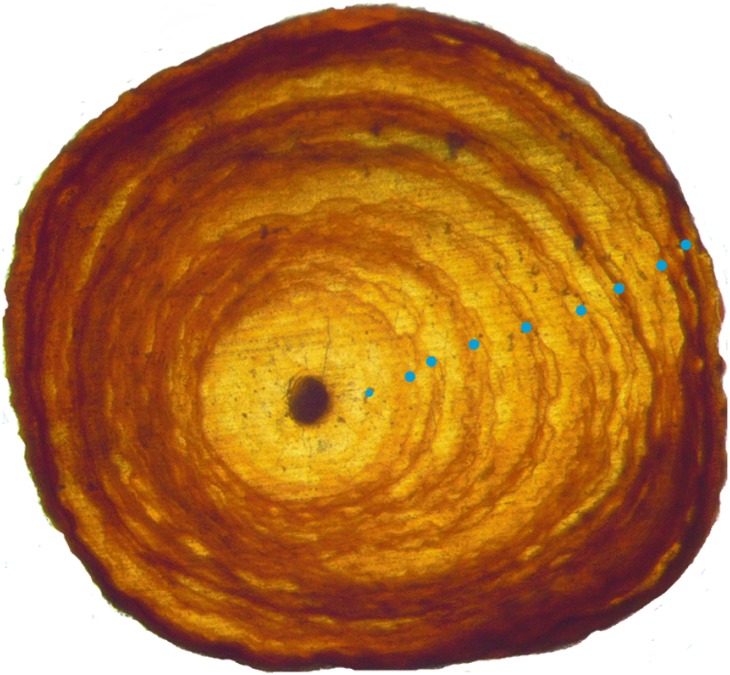
Growth rings in a basal cross section from a *Leptogorgia virgulata* colony under a compound microscope. Growth rings are denoted by the dots, and this colony was estimated to be 9 years old. This cross section is about 3 mm in diameter.

After air-drying colonies in the lab until completely dry, short (≈10 mm) pieces of the axial skeleton were cut from the base of each coral, and placed in a single 2 × 3 × 1 cm well within a silicon tray. Prior to embedding, the molds were sprayed with a silicon spray and left to dry. The epoxy solution was mixed for at least a minute, and then poured over the coral sections in the wells until it covered them completely. The epoxy resin blocks were left to dry for at least 8 h, and then sliced with a diamond bladed saw into sections of approximately 18 µm thickness (range 15–22 µm). Five consecutive sections of each basal piece were mounted on glass slides with crystal bond and photographed under a stereo-zoom microscope. Photos were then viewed in Adobe Photoshop to estimate age by counting annuli rings from the center outwards (Fig. 3). Preliminary analysis indicated that age estimates did not vary between sequential sections, and because the low quality of some sections made them unusable, we chose to analyze the best (i.e., clearest) section from each colony. Criteria for rings included either of two criteria (Mitchell, Dardeau & Schroeder, 1993): (1) A concentric band darker than the surrounding tissue, or; (2) A change in density or color of the axial rod in the inner region of the cross section. Growth rings were counted out to the growing edge of the coral, this edge not being counted as a ring unless it met the previous criteria.

The in-situ total length measurements were used to assign putative ages to in-situ corals via the Isermann-Knight Method using the age-length key generated from the collected corals (Ogle, 2016), described in the following growth methods section. The functions required to assign these ages are included in the FSA, dplyr, and nnet packages in R (Venables & Ripley, 2002; R Core Team, 2017; Ogle, 2016; Ogle, 2018; Wickham et al., 2018).

Mean ages of in-situ and collected corals were compared between sites using a One-way ANOVA (R function aov), and a Tukey HSD test was used to determine if any significant differences occurred between sites. Age distributions were compared between sites using two-sample KS tests with a Bonferroni adjusted critical *α* of 0.008 (R Core Team, 2017). Age rings in coral sections were independently counted by two observers to estimate bias (Grigg, 1974). If the values differed between readers the average was used. The presence of bias and the proportional agreement between readers was evaluated in R using age-bias plots and three symmetry tests, including McNemar, Evans and Hoenig, and Bowker (Ogle, 2016; Ogle, 2018). All three tests focus on whether an age-agreement table is symmetric around the main diagonal; however, the tests differ in how they gather the data for comparison. Each test produces estimates of the degrees of freedom, a chi-squared value, and a *P* value. The null hypothesis for these tests is that no asymmetry (or bias) occurs within the age estimates. The McNemar test uses a maximally pooled approach by adding squared values above and below the diagonal agreement line and determining if the sums are equal. It doesn’t take into account where the values are relative to the main diagonal other than above or below. In contrast, the Evans and Hoenig test uses a diagonally pooled approach, which tests for differences in values pooled from off-diagonals that are the same “distance” from the main diagonal. Bowker’s test calculates chi-squared without any pooling, and tests for differences between cells that are in the same relative positions above and below the main diagonal.

Precision of the data was evaluated using three indices: percent agreement (PA), average percent error (APE) and the average coefficient of variation (ACV). The APE assumes the standard deviation of age is proportional to the mean age for individual corals. The ACV is a measure of the dispersion of data points in a data series around the mean, or the ratio of standard deviation to the mean, and does not have the assumption mentioned for APE. For both APE and ACV lower values indicate higher agreement, with ACV <5% indicating high precision.

### Growth

Age-length keys, observed and smoothed, were constructed using the total length and age data from collected corals. These keys show the proportion of ages (ring counts) within bins of five cm total length. Observed age-length keys were constructed using raw data, whereas smoothed age-length keys use a multinomial linear regression to model proportions at each age for each length category. The predicted proportion of coral at age for any one length interval is influenced by both the data for that interval and age as well as by data for other intervals and ages—resulting in predicted values that follow a smooth curve. Coral growth rate was determined by examining the relationship between length and age, and fitting that age-length data to a von Bertalanffy growth model using the following equation:

E[L—t]=L_∞_(1 −e^−^^K^^(^^t^^−^^t^^0^^)^)

where E[L—t] is the expected or average length at age t, L_∞_ is the asymptotic average length, K is the Brody growth rate coefficient or exponential rate of approach to L_∞_ (yr^−1^), and t_0_ is the age when mean length is zero (model artifact) (Beverton & Holt, 1957). Confidence intervals for parameters in non-linear models, like the von Bertalanffy model, are best found through bootstrapping methods and not the model summary. To do this, we followed the methods of Ogle (2016) using the nlstools package (Baty et al., 2015). Von Bertalanffy models have been used to study the growth patterns of other gorgonian coral species (Grigg, 1974; Mistri & Ceccherelli, 1993; Mistri & Ceccherelli, 1994; Goffredo & Lasker, 2006; Munari, Serafin & Mistri, 2013), as well as corals with size-dependent growth (Chadwick-Furman, Goffredo & Loya, 2000; Goffredo, Mattioli & Zaccanti, 2004).

The functions required to construct age-length keys are included in the FSA, dplyr, and nnet packages in R (Venables & Ripley, 2002; R Core Team, 2017; Ogle, 2016; Ogle, 2018; Wickham et al., 2018). The functions required to perform growth analyses and bootstrapping methods in R are contained in the FSA and nlstools packages (Baty et al., 2015; R Core Team, 2017; Ogle, 2016; Ogle, 2018).

## Results

### Colony complexity

**Figure 4:**
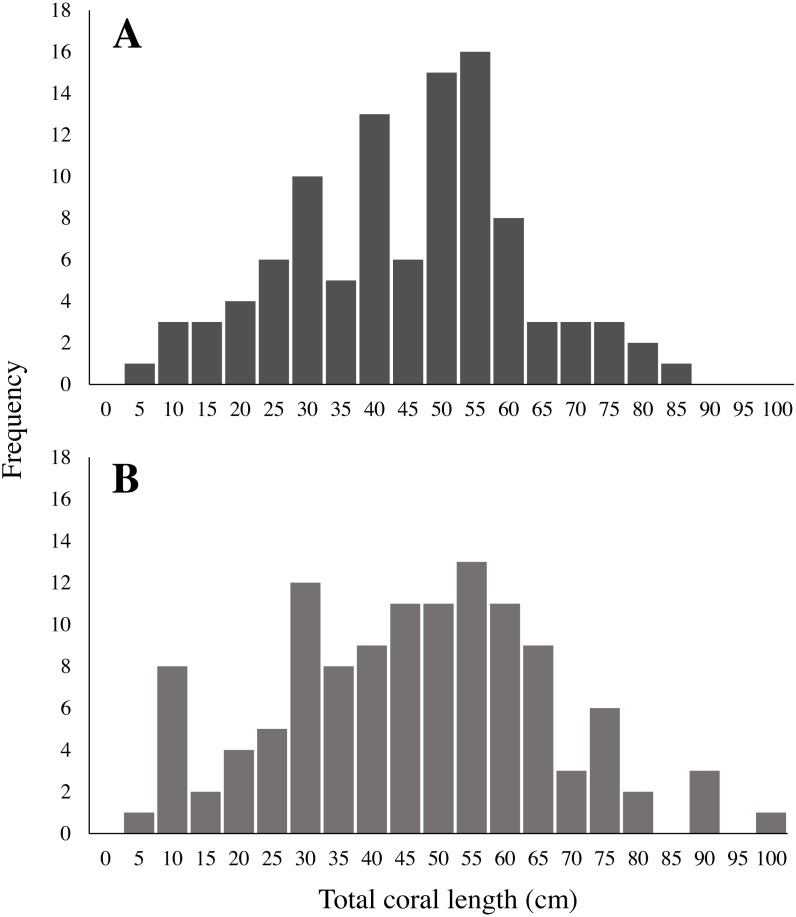
Length frequency of (A) collected and (B) in-situ measured *Leptogorgia virgulata* colonies. All four study sites are represented. (A) *N* = 102; (B) *N* = 119.

**Figure 5:**
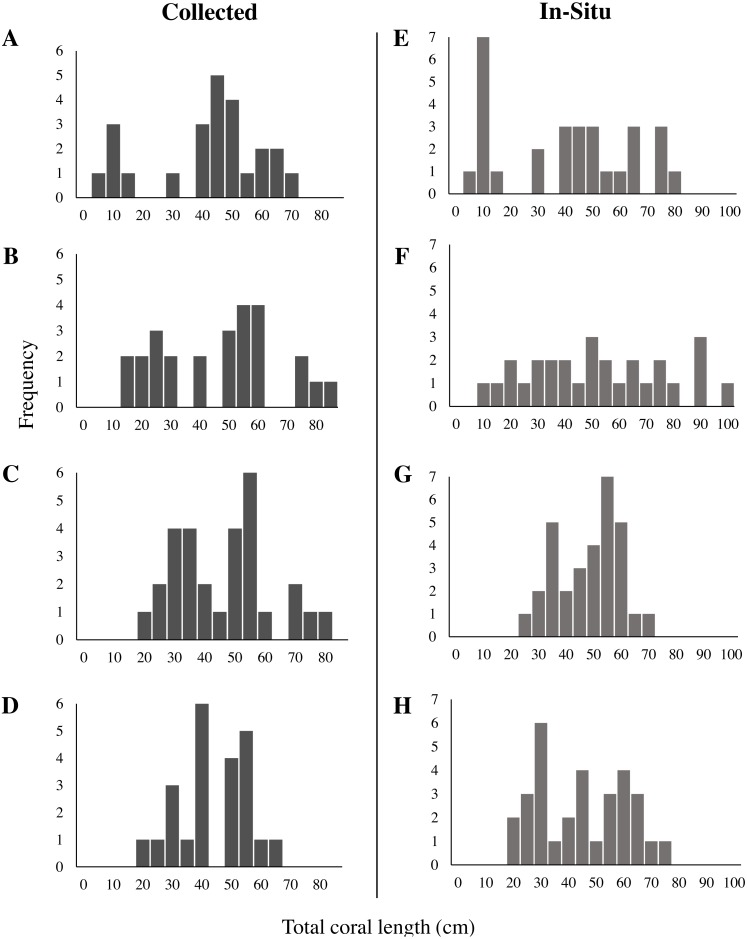
Length frequency of collected and in-situ *Leptogorgia virgulata* colonies at each study site. Four study sites: (A, E) Memorial Barge, (B, F) South Ledges, (C, G) Sussex Wreck, and (D, H) Boom Wreck. *N* = 24, 26, 29, and 23 respectively for collected corals, and *N* = 29, 28, 31, and 31 respectively for in-situ corals.

There was no significant difference in mean length and total length distributions between sites for both in-situ (One-way ANOVA, *F*(3, 115) = 1.84, *p* = 0.14; KS test × 6, all *p* > 0.008) and collected corals (One-way ANOVA *F* (3,98), *p* = 0.71; KS test × 6, all *p* > 0.008). Therefore, we pooled them together into a length-frequency figure containing all 119 corals measured in-situ and the 102 corals collected (Fig. 4). The mean length of in-situ sea whips was 48.6 ± 1.8 cm (mean ± SE), with total length ranging from 7.5 cm to 100.2 cm (Fig. 4), and 50% of in-situ sea whips were in the range of 33.7–61.8 cm. The mean length of collected sea whips was 46.9 ± 1.7 cm. Total length ranged from 8.3 cm to 85.3 cm, with 50% of corals in the range of 34.2–56.4 cm (Fig. 4). Total length frequency of both collected and in-situ sea whips per site are illustrated in Fig. 5. No significant differences in the bifurcation (R_b_) ratios of *L. virgulata* were found between sites (One-Way ANOVA, *F*(3, 97) = 0.0597, *p* = 0.81) (Table 2)*.* The average R_b_ ratios of approximately 3 for all sites indicated that for each branch of a given order, there are approximately three branches in the next lower order. For example, for every tertiary branch there are three secondary branches, and for every secondary branch there are three primary branches. There was also no significant difference found between tributary to source (T/S) ratios in primary (One-way ANOVA, *F*(3, 100) = 3.317, *p* = 0.072) and secondary (One-way ANOVA, *F*(3, 97) = 0.348, *p* = 0.556) branches between sites (Table 2).

### Age analysis

**Figure 6:**
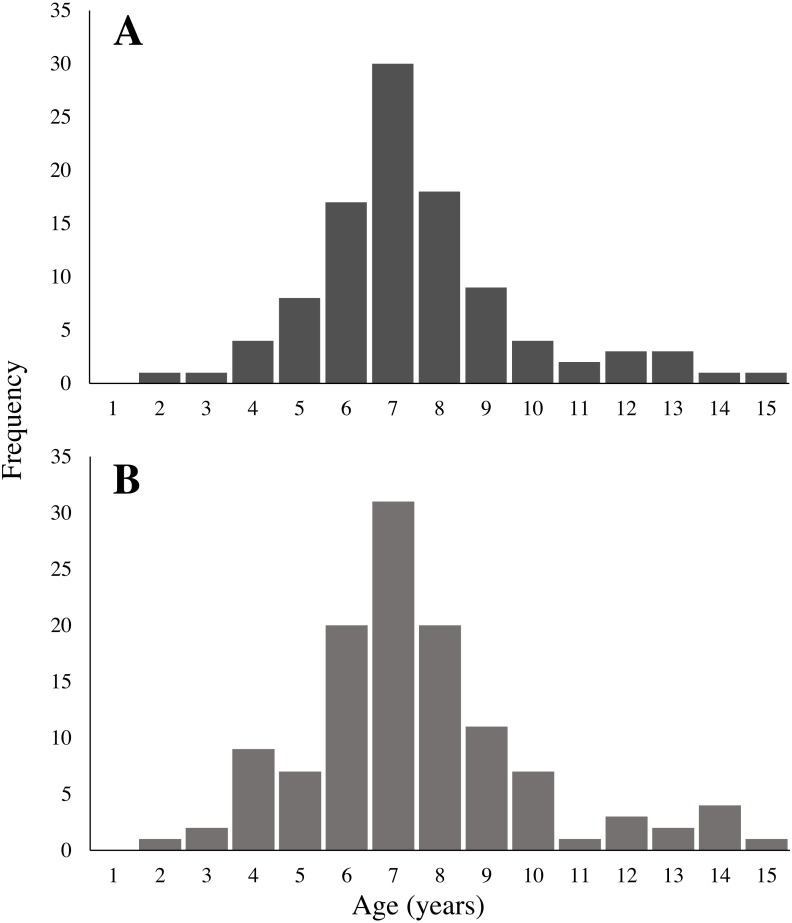
Age frequency of (A) collected and (B) in-situ * Leptogorgia virgulata* colonies. All four sites are represented. (A) *N* = 102; (B) *N* = 119.

**Figure 7:**
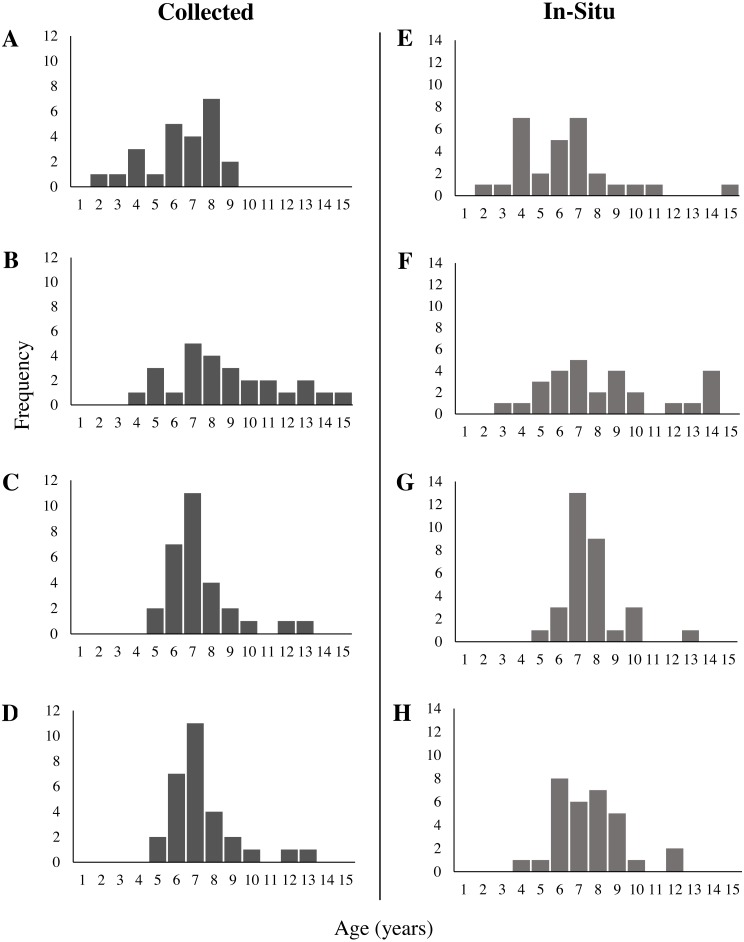
Age frequency of collected and in-situ *Leptogorgia virgulata* colonies at each study site. Four study sites: (A, E) Memorial Barge, (B, F) South Ledges, (C, G) Sussex Wreck, and (D, H) Boom Wreck. *N* = 24, 26, 29, and 23 respectively for collected corals, and *N* = 29, 28, 31, and 31 respectively for in-situ corals.

Neither mean age nor age distribution of collected corals differed significantly between sites (One-way ANOVA, *F* (3,100), *p* = 0.55; KS test × 6, all *p* > 0.008). Therefore, data for collected corals from all four sites were grouped together for further analysis (Fig. 6). Estimated age of collected sea whips ranged from 2 to 15 y, with 50% in the range of 6 to 8 y. The distribution of assigned ages for in-situ corals was not significantly different between sites (KS test × 6, all *p* > 0.008), however there was a significant difference in their mean assigned ages (One-way ANOVA, *F* (3,115), *p* = 0.013). Further analysis with a post hoc Tukey HSD test showed that mean age differed significantly ( *p* = 0.008) between the Memorial Barge (6.3 y) and South Ledges (8.3 y) sites, with mean ages for other sites not significantly different. Assigned ages for in-situ corals >85 cm are also most likely underestimated (see Growth section below). Age frequency of both collected and in-situ sea whips per site are illustrated in Fig. 7. Three tests of symmetry showed no systematic bias between readers (McNemar *p* = 0.64, Evans and Hoenig *p* = 0.64, Bowker *p* = 0.38) (Table 3). *P*-values > 0.05 for each test indicate that the null hypothesis has not been rejected, therefore no significant asymmetry was observed. Indices of precision show that percent agreement (PA) between readers was 82.35%, with the remaining 18% differing by only 1 year. The average percent error (APE) was 1.2%, and the average coefficient of variation (ACV) was 1.7%, therefore our age counts can be considered precise (ACV < 5%).

### Growth

**Figure 8:**
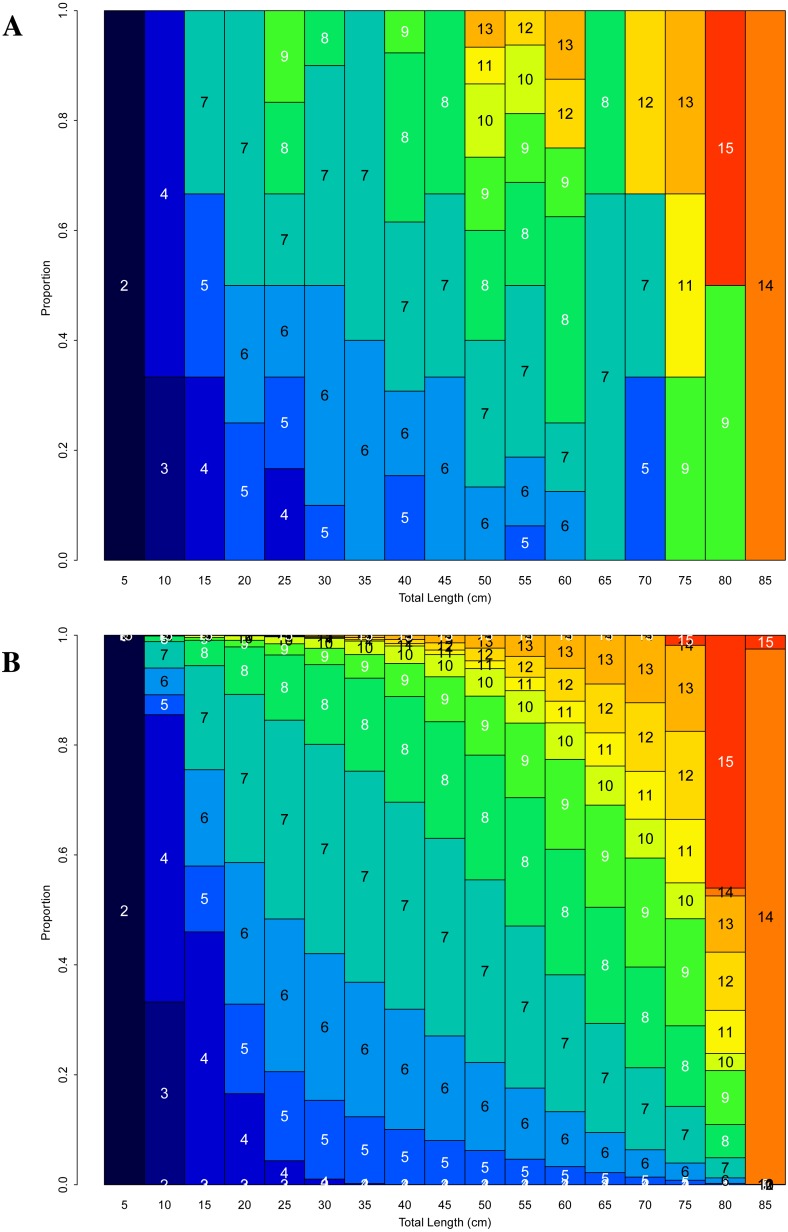
(A) Observed and (B) smoothed age-length keys for * Leptogorgia virgulata* colonies. All four study sites are represented. (A) *N* = 102. Colored blocks represent the proportion of corals at that age within the 5 cm total length bin.

The age-length keys generated from the pooled coral data show the trend of age increasing with total coral length (Fig. 8). In the observed age-length key there are portions that seem to contradict the overall trend, such as the age 7 and 8 corals in the 65 cm interval following the age 9–13 corals in the three previous intervals. This is a common issue with observed age-length keys, and can be due to highly variable ages within a length interval and small sample sizes in some length intervals. The smoothed age-length key, which applies a multinomial logistic regression model fit to all length intervals and ages, addresses those issues and more clearly shows the trend of increased age with total coral length (Fig. 8).

**Table 2:** Colony complexity of *L. virgulata* samples from four sites in the Mid-Atlantic Bight. Bifurcation ratios (R_b_) are the number of branches of a given order to the number of branches of the next higher order, and tributary to source ratios (T/S) are the number of tributary branches vs. the number of source branches. T/S ratios were calculated for both 1° and 2°, primary and secondary, branches.

Site	*N*	R_b_	T/S ratios	
			1°	2°
Memorial barge	24	3.0	0.60	0.43
South ledges	26	2.8	0.52	0.39
Sussex wreck	29	2.9	0.41	0.53
Boom wreck	23	2.9	0.48	0.44
Significance		NS	NS	NS

**Notes.**

N number of colonies NS not significant (One-way ANOVA, *P* > 0.05)

**Figure 9:**
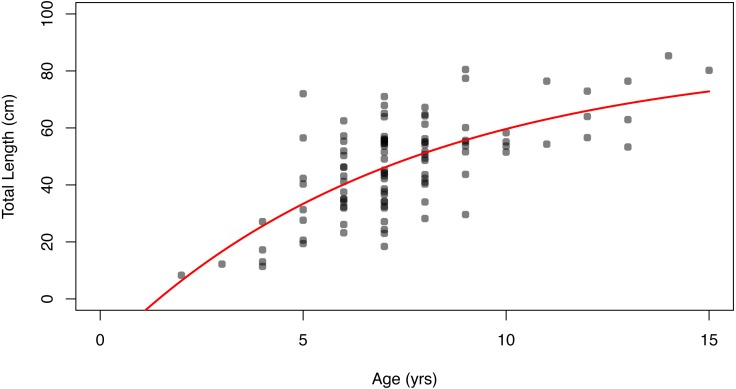
The von Bertalanffy growth model for *Leptogorgia virgulata* colonies collected from all four study sites.

Coral growth was determined by relating age estimates to total coral length in a von Bertalanffy growth model (Fig. 9), using all 102 corals collected. Our model demonstrates size-dependent growth, and the parameters were calculated to be: L_∞_ = 86.1 cm (95% CI [66.7–219.6], *P* = 0.000003), *K* = 0.14 yr^−1^ (0.028–0.26, *P* = 0.029), and *t*_0_ = 1.44 y (−1.37–2.53, *P* = 0.098). This results in the equation E[L—t] (cm) = 86.1(1 −e ^−^^0.14^^(^^t^^−^^1.44^^)^)

where E[L—t] represents length at age t. Therefore, the curve reaches an asymptotic mean length of 86.1 cm at the exponential rate of approach of 0.14 yr^−1^, with corals about 20 years in age. The age at which mean length is 0, or t_0_, is 1.44 years. However, t_0_ is a modeling artifact and has little biological significance. The maximum observed length of in-situ corals (100.2 cm) was greater than the largest length category in the age-length keys (85 cm). Furthermore, the value of L_∞_ = 86.1 is intended to estimate the mean size of the largest corals, not the maximum size. Therefore, when assigning ages to the in-situ corals the last length category was treated as all-inclusive. Subsequently, the ages of in- situ corals >85 cm are most likely underestimated.

## Discussion

The mean height of collected sea whips in our study was 46.9 ±1.7 cm, with a total length distribution of 8.3 cm to 85.3 cm, and 50% of corals in the range of 34.2 to 56.4 cm. In contrast, *L. virgulata* colonies studied by Mitchell, Dardeau & Schroeder (1993) in the Gulf of Mexico had a mean size of 18.9 cm and none exceeded 32.5 cm in height. Mitchell, Dardeau & Schroeder’s (1993) study site was only 1–1.5 m in depth and exposed to more frequent wave action and subsequent sand scouring than our sites which were at depths of 17–26 m. This could have prevented coral growth to larger heights. However, strong and tall communities of gorgonian corals have been documented on habitats exposed to strong surf (Kinzie, 1973; Birkeland, 1974; Sánchez, Díaz & Zea, 1997; Gomez et al., 2014).

### Colony complexity

The average bifurcation ratio (R_b_) equaled approximately 3 for all study sites, indicating that for each branch of a given order there are approximately three branches in the next lower order. This coincides with the R_b_ of 3.1 that Mitchell, Dardeau & Schroeder (1993) found for the sea whips in their study, suggesting this may be a characteristic of the species. In many arboreal gorgonians, the distal portion of first order branches include many young, nonreproductive polyps while older, reproductive polyps are more numerous on secondary and tertiary branches (Brazeau & Lasker, 1988).

There was no significant difference found between tributary to source (T/S) ratios in primary and secondary branches between sites. Larger primary T/S ratios at Memorial Barge and South Ledges indicate that there are more primary than secondary tributary branches in the colonies at each site, which may explain their “bushier” appearance, however it doesn’t contribute to changing the overall colony complexity as indicated by the bifurcation ratio. The lower primary T/S ratios at the deeper Sussex and Boom Wreck sites indicate fewer accessory (tributary) branches, but the difference was not significant. This pattern was also seen in Brazeau & Lasker’s (1988) study, which looked at the colony complexity of two arborescent gorgonian species, *Plexaura homomalla* and *Plexaura flexuosa,* at shallow and deep sites*.* They found primary and secondary T/S ratios to decrease significantly with depth, leading to the “bushier” appearance of corals at the shallow site (Brazeau & Lasker, 1988). Though the differences were not significant in our study, the reoccurrence of the pattern suggests that the colony complexity of arborescent gorgonian corals, like *L. virgulata*, can change with depth. In corals containing zooxanthellae, this change in colony morphology could serve to reduce self-shading at lower light levels (Brazeau & Lasker, 1988). Like other cold-water corals, however, *L. virgulata* lacks zooxanthellae (Roberts, Wheeler & Freiwald, 2006). Therefore, morphological plasticity in this species could be due to other depth-related factors, such as wave stress, current intensity, food density, and predation (Lasker, Gottfried & Coffroth, 1983; Dai & Lin, 1993; West, Harvell & Walls, 1993; Sánchez, Díaz & Zea, 1997).

**Table 3:** Tests of symmetry between age estimates made by readers 1 and 2.

Test	df	*χ*^2^	*P*
McNemar	1	0.22	0.637
Evans and Hoenig	1	0.22	0.637
Bowker	7	7.47	0.382

**Notes.**

*df* degrees of freedom *χ*^2^ chi-square value

*N* = 102.

### Age analysis

Our sea whip samples had an age range of 2 to 15 y, with 50% in the range of 6 to 8 y. Agreement on ring counts between readers was high, and there was no systematic bias displayed in ageing by either reader. There was more disagreement at greater ages, most likely because rings towards the outer edge of the coral tend to be smaller in width and closer together (Grigg, 1974), making it harder to distinguish between them. However, estimates never differed by more than one year.

Despite our systematic approach to sampling and *L. virgulata*’s annual reproductive season (Adams, 1980), we observed very few juveniles and a large proportion of middle-age colonies. This is similarly expressed in the size frequency of corals collected and those measured in-situ, which show fewer colonies in the smaller size classes and a larger proportion in the mid-size classes. The dominance of medium-aged *L. virgulata* colonies is consistent with episodic recruitment, with the high frequency of middle-age colonies representing a past “pulse” in recruitment (Munari, Serafin & Mistri, 2013). This episodic recruitment may be due to environmental factors influencing the mortality and settlement of coral larvae, newly settled recruits, and/or juvenile colonies. Smaller gorgonian corals have been observed to have higher mortality rates than those in larger size classes (Grigg, 1977; Gotelli, 1991; Gomez et al., 2014). Yoshioka (1994) observed this trend in gorgonian *Pseudopterogorgia* spp. populations, where larger colonies had a high (96% y^−1^) and constant survivorship compared to the low (62% y^−1^) and variable survivorship in smaller colonies. This resulted in episodic variations in *Pseudopterogorgia* population size frequencies. Gotelli (1991) noticed high variation in the number of *L. virgulata* recruits on a monthly scale, as well as low juvenile survivorship. Predation does not appear to be a major factor behind *L. virgulata* juvenile mortality (Patton, 1972). Episodic recruitment could potentially explain the significantly different mean ages of the in-situ corals at Memorial Barge and South Ledges. Differential recruitment between the sites could lead to this difference in mean age.

Episodic recruitment can also indicate the instability of the species’ environment, as the more variable the environment the more irregular the age structure of a respective population will be (Grigg, 1975). One such environmental factor affecting the observed age-frequency in our study could be storm events. Though their effect on gorgonian communities can be unpredictable and highly variable, large storms can produce strong currents and wave action resulting in the burial, sand scouring, breakage, and detachment of corals (Yoshioka & Yoshioka, 1987). Detachment and abrasion were a major cause of mortality in Grigg’s (1977) study examining populations of the branching gorgonians *Muricea californica* and *Muricea fruticosa,* with colonies able to withstand detachment by strong currents until they reached a threshold height and size*.* Gotelli (1988) concluded that high sediment concentrations limit recruitment of *L. virgulata* larvae by restricting settlement sites, and that sand was an important source of juvenile mortality due to the burial or damage of young or newly settled individuals. Larger, adult colonies appear to be tolerant of heavy sediment loads, perhaps due to their established holdfast and additional height (Williamson et al., 2011). Storm events could also be the reason why our age frequency and distribution differed from that of Mitchell, Dardeau & Schroeder (1993), whose study population of *L. virgulata* experienced two hurricanes within 8 years of the project. The Memorial Barge site is the shallowest and northern-most of all our sampled sites. Wind records from NOAA Buoy 44009 (Delaware Bay), located 26.4 km to the northeast, indicate that a major storm in November of 2009 caused wave heights >8 m for two days; this event could have removed sea whip colonies and other organisms from the site, opening settlement areas for new recruits, which would explain the age distribution at that site. Hurricane Sandy (Oct 22, 2012) caused wave heights of 7.4 m for one day; maximum wave heights in 2014-2015 were <6 m, but in January of 2016, two consecutive days with winds >20 m ⋅s^−1^ and waves >8.4 m occurred, which destroyed both the wave height and wind speed sensors.

In addition to storm activity, we speculated as to how variation in depth and water temperature may have led to the different age distribution and frequency found between our study and Mitchell, Dardeau & Schroeder (1993). There are contradictions about how depth, and associated flow velocities, affect gorgonians. Dai & Lin (1993) state that gorgonian corals feed optimally at moderate flow velocities, and Lasker, Gottfried & Coffroth (1983) found that feeding rates of both gorgonian species studied were lower at 29 m than 17 m, but they could capture food at both. Contrastingly, Sánchez, Díaz & Zea (1997) found greater gorgonian diversity and cover to occur on mid to low relief sections of fore reef terraces, where wave turbulence, flow rate, and turbidity is higher. When looking at temperature, Rossi et al. (2004) found the digestion time of *Leptogorgia sarmentosa* to increase as temperature decreased, which would lead to slower growth rates in colder temperatures. However, Previati et al. (2010) found the respiration rate of four gorgonian species to decrease at temperatures higher than 18−20 °C, indicating a reduction of metabolic activity at higher temperatures. At a sustained 3 day temperature of 25 °C, the three azooxanthellate corals exhibited the first signs of necrosis. With the coastal waters of the Gulf of Mexico reaching average temperatures higher than this in the summer months, the survival of *L. virgulata* colonies in Mitchell, Dardeau & Schroeder (1993) could have been negatively impacted, resulting in the different age frequency and distribution observed.

Another factor preventing regular recruitment in the population studied by Grigg (1977) was the lack of available hard substrata for settlement, a determining factor of recruitment for many gorgonians (Kinzie, 1973). Therefore, recruitment may be regulated by space limitation in habitats where hard substrata is completely occupied, or covered in a soft sediment layer. Coral larvae that do manage to settle may then have to compete with other sessile organisms, and risk mortality by overgrowth (Gomez et al., 2014). On our study sites, the hard substrata of the artificial reef were often covered in beds of mussels, sponges, and the encrusting star coral *Astrangia poculata*, as well as an occasional layer of fine sediment. Thus new coral recruits may find stiff competition for available substrata on these sites crowded with other organisms. Years of high *L. virgulata* recruitment may result after a removal or mortality event affects established sessile organisms or sediment layers, clearing space for coral settlement. This was the case in Grigg’s (1977) study, where occasional periods of exposed hard bottom due to shifting sediments resulted in fluctuating recruitment of *Muricea.* Large year classes resulted from years of heavy recruitment after substrata exposure. Similarly, large mussels covered one of our study sites (Memorial Barge) in 2016, but when that site was revisited in 2017, the mussels were scarce and much smaller; by 2019, the site was again covered with a deep layer of small mussels. This suggests that mussels may have been removed by winter storms between visits, and replaced by new recruits, which could have direct impacts on coral recruitment. This conclusion is supported by wind data. Unfortunately, NOAA Data Buoy 44009 was not repaired until January 2017, so there are no wind or wave height data during the fall of 2016, although records from Buoy OCIM2 (Ocean City Inlet) show winds reaching 17.2 m ⋅s^−1^ on October 9, 2016, one week after we sampled the site. Wave heights associated with winds >17.5 m at NDBC 44009 were typically >4 m.

### Growth

The generated age-length keys show the overall pattern of coral size increasing with age. Anomalies to the trend found in the observed age-length key could be due to variable ages and/or small sample sizes in some length intervals, which are common issues within these keys. The multinomial logistic regression applied in the smoothed age-length key addresses those issues and better shows the trend of increased age with total coral length. Gotelli (1991) also found a correlation between *L. virgulata* colony size and age, with considerable variation in the size of older individuals. Variation of growth rate between colonies, and the subsequent variation in length for colonies of similar ages, may be due to persistent intrinsic differences, minor differences in food supply related to position on the reef, or other differences in the microhabitat (Grigg, 1974). This variation could also be explained by the potential net negative growth of older colonies whose branches have experienced damage or tip removal, reducing their total length (R Wenker and B Stevens, pers. obs., 2017 and 2018; Grigg, 1974; Mistri & Ceccherelli, 1994; Rossi, Gili & Garrofé, 2011; Cupido et al., 2012).

For organisms with indeterminate growth and variation of size within an age class, it has been recommended to use population dynamic models based not only on age, but on simultaneous analyses of size and age (Kirkpatrick, 1984; Hughes, 1984; Hughes & Connell, 1987). We accomplished this using a von Bertalanffy growth model illustrating *L. virgulata* size at age. According to this growth function, *L. virgulata* reaches maximum individual length at approximately 20 years of age. Gorgonian corals tend to grow toward a theoretically high size asymptote, with their size and lifespan then being limited ecologically (Grigg, 1974; Mistri & Ceccherelli, 1993; Mistri & Ceccherelli, 1994; Goffredo & Lasker, 2006; Munari, Serafin & Mistri, 2013)—a trend that appears to apply to *L. virgulata*. Other constraints on size may be physical, like the biomechanics of a coral skeleton with a highly branched structure (Chadwick-Furman, Goffredo & Loya, 2000). Mitchell, Dardeau & Schroeder (1993) produced a growth function for *L. virgulata* using a Walford plot, where a K parameter equal to 0.094 y^−1^ can be derived from the slope. This is a lower rate of approach compared to our K value of 0.14 y^−1^, suggesting the sea whips in our study approach L_∞_ at a slightly faster rate.

With no corals over the age of 15, and 50% in the range of 6 to 8 y, our study sites are dominated by middle-age colonies. Our curve seems to be slightly below the rapid, two-year initial growth rate of *L. virgulata* which Adams (1980) observed, where age-2 corals averaged 14 cm in height. However, that study mimicked water conditions of the Gulf of Mexico, so the warmer temperatures could have potentially increased *L. virgulata* growth rate in comparison to our study. This difference could also be due to sample size limitation, as we found only one coral aged 2 y ( eight cm), and no corals younger than that. This colony displayed a growth rate of four cm per year, which is lower than the seven cm per year calculated by Adams (1980) for age-2 colonies. In general, younger corals were scarce in our samples, with only fourteen corals under 6 y out of the 102 collected. This could have affected the t_0_ model parameter, age at length 0 cm. Additionally, only 22.5% of the corals collected were over 8 y, which could potentially alter the rate of approach (K) to L_∞_.

The presence of mostly middle-age colonies at our study sites implies that adult survivorship is high for these populations. In Gotelli’s (1991) study of *L. virgulata,* the population growth rate (growth rate measured as the proportion of colonies in size class *i* that grew to size class *i* + 1 in the next month) was close to 0.0. Thus, it was clear that adult survivorship was more important to population growth than either recruitment or fecundity. One reason for this could be fluctuating juvenile mortality (Gotelli, 1988; Gotelli, 1991; Yoshioka, 1994). Therefore, the presence of many middle-age adults at our sites could be considered beneficial in terms of maintaining overall population structure and growth. However, Gotelli (1991) did mention that recruitment was important in terms of stabilizing the population growth of *L. virgulata*.

In the Delmarva region of the MAB there is evidence of threats to adult survivorship, and subsequently the population stability of *L. virgulata* populations. Schweitzer, Lipcius & Stevens (2018) observed commercial fishing activities at 3 sites in this region, and found that 50% of the commercial fishing traps observed came into contact with benthic epifauna, including sea whips, upon retrieval. As a result, sea whips could be damaged or experience breakage, reducing their overall structural complexity and density. Our study sites are not commercially fished, however they are fished recreationally. Observed damage to sea whips at these sites includes fishing lines entangled in biotic overgrowth attached to the corals, lines restricting coral branches, lines cutting into coral tissue, and damaged and stripped tissue in general. Future work is needed to examine to what degree fishing damages *L. virgulata* or affects its survival; however, numerous studies have highlighted the negative impacts fishing can have on cold-water corals (Van Dolah, Wendt & Nicholson, 1987; Fosså, Mortensen & Furevik, 2002; Freiwald et al., 2004; Roberts, Wheeler & Freiwald, 2006; Watling et al., 2011).

## Conclusions

This study currently represents the only measure of colony complexity, age, and growth for *L. virgulata* on artificial reefs in the mid-Atlantic region, and will be a useful reference to study changes over time and/or long-term population trends. However, the number of sites observed and corals analyzed may represent a fraction of the total number in this region (Office of Coast Survey, 2019). Therefore, more research into the location and biology of *L. virgulata* in this region is necessary to better verify the coral characteristics presented in our study. While ageing corals via growth ring counts may be a more accurate technique, the growth function generated in our study could be used in the field to estimate age-frequency without removal of specimens. Additionally, measuring in-situ changes in total coral length via tagging studies would give more insight into growth rates of *L. virgulata*. They could also be used to improve our understanding of the capacity for, and rates of recovery of sea whip populations after damage or removal by either human or natural disturbance. The evidence for episodic recruitment of *L. virgulata* shown in this study suggests that they do not recruit on a regular annual basis, and good recruitment years may only occur at intervals of a decade or longer. Thus, any corals that are damaged or removed due to disturbance by human or natural events may require decades to recover. Changes in fishing patterns, storm events, or climate change may exacerbate or change this pattern. Presently, the most conspicuous human disturbances to *L. virgulata* in the mid-Atlantic result from trap and hook and line fishing. However, the planned development of offshore wind-power energy areas (Maryland Energy Administration, 2019) may also impact coral populations in the future. In addition, our study only looked at recreationally fished sites because most natural bottom sites in our area (and those targeted by commercial fishers) were at depths >25 m, beyond the range of air-based scuba. Studies using cameras and ROVs (Schweitzer, Lipcius & Stevens, 2018) show that *L. virgulata* is common on natural reefs targeted by commercial fishing at depths of 20–33 m, and visual observations do not indicate any difference between colonies at those sites and the ones we studied. Therefore, it would be beneficial for future studies to examine sea whips at commercially fished sites in order to compare them. Regarding natural disturbance, storm events are most likely the biggest source of damage, and the frequency and intensity of storm events should be considered for long-term modeling, management, and habitat protection. Finally, because our study sites only included artificial reef sites, it would be valuable to examine sea whips on natural hard-bottom to determine if there are any differences between corals on artificial and natural reefs. Once the information regarding population distribution, biology, and trends in these waters have been more thoroughly documented, the development of population models for coral communities would be beneficial.

##  Supplemental Information

10.7717/peerj.8372/supp-1 Supplemental Information 1 Smithsonian Donation Sea whip Catalogue Numbers

## Additional Information and Declarations

### Field Study Permissions

The following information was supplied relating to field study approvals (i.e., approving body and any reference numbers): Field collection of coral specimens was approved through a federal exemption by the National Oceanic and Atmospheric Administration via a letter of acknowledgement.

### Data Availability

The following information was supplied regarding data availability: Data and code are available at Zenodo: Wenker, Rebecca, & Stevens, Bradley. (2019, September 16). Sea whip coral in the Mid-Atlantic Bight: Colony complexity, age, and growth. Zenodo. DOI: 10.5281/zenodo.3417694. Rebecca Wenker, & Bradley Stevens. (2019). Sea whip coral Leptogorgia virgulata in the Mid-Atlantic Bight: Colony complexity, age, and growth [Data set]. Zenodo. DOI: 10.5281/zenodo.3547945.
